# Early statin use in ischemic stroke patients treated with recanalization therapy: retrospective observational study

**DOI:** 10.1186/s12883-015-0367-4

**Published:** 2015-07-30

**Authors:** Jihoon Kang, Nayoung Kim, Tae Hwan Park, Oh Young Bang, Ji Sung Lee, Juneyoung Lee, Moon-Ku Han, Seong-Ho Park, Philip B. Gorelick, Hee-Joon Bae

**Affiliations:** Department of Neurology, Samsung Changwon Hospital, Sungkyunkwan University School of Medicine, Changwon, Korea; Department of Neurology, Cerebrovascular center, Seoul National University Bundang Hospital, Seoul National University, Seongnam, Korea; Department of Neurology, Seoul Medical Center, Seoul, Korea; Department of Neurology, Stroke and Cerebrovascular center, Samsung Medical Center, Sungkyunkwan University School of Medicine, Seoul, Korea; Clinical Research Center, Asan Medical Center, Seoul, Korea; Department of Biostatistics, Korea University College of Medicine, Seoul, Korea; Department of Translational Science and Molecular Medicine, Michigan State University College of Human Medicine & Saint Mary’s Health Care at Mercy Health, Grand Rapids, Michigan USA

**Keywords:** Stroke, Recanalization, Statin, Stenosis and occlusion

## Abstract

**Background:**

We aimed to determine whether early statin use following recanalization therapy improves the functional outcome of ischemic stroke.

**Methods:**

Using a prospective stroke registry database, we identified a consecutive 337 patients within 6 h of onset who had symptomatic stenosis or occlusion of major cerebral arteries and received recanalization therapy. Based on commencement of statin therapy, patients were categorized into administration on the first (D1, 13.4 %), second (D2, 20.8 %) and third day or later (D ≥ 3, 15.4 %) after recanalization therapy, and no use (NU, 50.4 %). The primary efficacy outcome was a 3-month modified Rankin Scale score of 0–1, and the secondary outcomes were neurologic improvement, neurologic deterioration and symptomatic hemorrhagic transformation during hospitalization.

**Results:**

Earlier use of statin was associated with a better primary outcome in a dose-response relationship (*P* for trend = 0.01) independent of premorbid statin use, stroke history, atrial fibrillation, stroke subtype, calendar year, and methods of recanalization therapy. The odds of a better primary outcome increased in D1 compared to NU (adjusted odds ratio, 2.96; 95 % confidence interval, 1.19–7.37). Earlier statin use was significantly associated with less neurologic deterioration and symptomatic hemorrhagic transformation in bivariate analyses but not in multivariable analyses. Interaction analysis revealed that the effect of early statin use was not altered by stroke subtype and recanalization modality (*P* for interaction = 0.97 and 0.26, respectively).

**Conclusion:**

Early statin use after recanalization therapy in ischemic stroke may improve the likelihood of a better functional outcome without increasing the risk of intracranial hemorrhage.

**Electronic supplementary material:**

The online version of this article (doi:10.1186/s12883-015-0367-4) contains supplementary material, which is available to authorized users.

## Background

Recanalization therapy for acute ischemic stroke aims to restore the patency of occluded cerebral arteries and subsequent brain reperfusion [1]. Since the introduction of intravenous (IV) thrombolysis, additional attempts have been made with a new generation of thrombectomy devices that show much higher successful recanalization rates [2, 3].

Unfortunately, this improvement is in discord with clinical outcome; only ~ 30–40 % of ischemic stroke patients had good functional outcome irrespective of treatment modality [2–4]. The discrepancy between recanalization and functional outcome may be attributed, at least in part, to no-reflow phenomenon, reperfusion injury, and re-occlusion [4, 5]. A number of studies have been designed to improve outcome by preventing these adverse events [6, 7]. Most recently, early aspirin use after IV thrombolysis was investigated for the prevention of secondary thrombosis [8]. It did not improve outcome but increased bleeding.

Pleiotropic effects of statin might be beneficial for improving functional outcome and preventing intracranial hemorrhage in patients who have undergone recanalization therapy [9, 10]. In experimental stroke models, early statin administration enhances thrombolysis, augments antithrombotic responses, increases cerebral blood flow, and decreases matrix metalloproteinase-9 (MMP-9) levels [11–14]. These actions predispose the prevention of re-occlusion and improve the prospects for brain perfusion. Until now, the effect of statin treatment following recanalization therapies has been examined once in patients who have received IV thrombolysis [15]. The effects of statin may be maximized in patients who present with acute steno-occlusion of the major cerebral arteries and are treated successfully with recanalization therapy, including endovascular approaches.

Therefore, we aimed to determine whether early statin use in patients with acute symptomatic steno-occlusion of major cerebral arteries who are treated with recanalization therapy is associated with a better 3-month functional outcome irrespective of recanalization modality. Associations of early statin use were further analyzed in relation to the following other neurologic outcomes obtained during hospitalization: stroke recurrence, neurologic improvement, neurologic deterioration, and symptomatic hemorrhagic transformation. Furthermore, we studied the effect of statin dose and the heterogeneity of the early statin effect by stroke subtype and recanalization modality.

## Methods

### Institutional review board approval and patient consent

This study was approved by the Seoul National University Bundang Hospital Istitutional Review Board (SNUBH IRB) with waiver of informed consent because of its minimal risk and retrospective nature.

### Study participants

Based on a prospective stroke registry database [16], a consecutive series of patients 1) who were admitted to Seoul National University Bundang Hospital for acute ischemic stroke within 6 h of symptom onset between March 2004 and September 2011, 2) who underwent recanalization therapy and 3) who had symptomatic stenosis (>50 %) or occlusion of a major cerebral artery at initial angiographic evaluation were identified. According to the institutional stroke image protocols, patients who were potentially eligible for recanalization therapy underwent computed tomographic angiography (CTA) or magnetic resonance angiography (MRA) at presentation. In this study, the cerebral arteries of interest were the internal carotid artery (ICA), middle cerebral artery (MCA), anterior cerebral artery (ACA), posterior cerebral artery (PCA), basilar artery (BA), and vertebral artery (VA). Patients without a 3-month modified Rankin Scale (mRS) score were excluded.

### Data collection and outcome measures

Review of electronic medical records and the registry database provided clinical information on demographic factors, baseline stroke severity as measured by the National Institute of Heath Stroke Scale (NIHSS), vascular risk factors, and acute stroke management. Recanalization modalities were classified into the following types: IV thrombolysis (IV-only), intra-arterial treatment (IA-only), and combination of IV and IA treatments. IA treatment included IA use of chemical thrombolytic agents, clot maceration by multiple passages of microcatheter/microwire [17], and mechanical thrombectomy using devices such as the Penumbra system and the Solitaire. Stroke subtype was assigned by a vascular neurologist according to the Trial of Org 10172 in Acute Stroke Treatment (TOAST) [18] criteria and validated at a weekly stroke registry meeting by consensus.

Premorbid statin use, defined as receiving statin therapy within 1 week before stroke onset, was ascertained by patient or proxy interview. Timing of statin administration after recanalization therapy, specific statin type, and initial dose were ascertained by review of the electronic medical records. Commencement of statin therapy was categorized according to the following scheme: statin administration on the first (D1), second (D2), and third day or later (D ≥ 3) of hospitalization, and no statin therapy (NU). Since statin was used in several forms and doses, we substituted a specific dose of a specific form of statin with an equivalent dose of atorvastatin (10 mg or less, 20 mg, 40 mg and 80 mg) [19, 20]. The atorvastatin equivalent doses were then categorized into no use, low (less than atorvastatin equivalent dose of 40 mg) and high dose (40 mg or more) [21].

After IRB approval, we prospectively collected NIHSS scores at baseline and on the second and seventh day of hospitalization or at discharge, as well as the 3-month mRS scores, as part of an institutional quality-of-care monitoring program for hospitalized stroke patients. The primary efficacy outcome of the study was good functional outcome at 3 months (mRS score of 0–1) [22]. A favorable outcome, defined as a 3-month mRS score of 0–2, was a secondary outcome.

Other secondary outcomes included neurologic improvement, neurologic deterioration, and ischemic stroke recurrence during hospitalization. Neurologic improvement was defined as a reduction in total NIHSS score of ≥4 points from baseline to discharge or a NIHSS score of 0–1 at discharge. Neurologic deterioration was defined as an increase in a total NIHSS score of 4 or more points from baseline [23]. Ischemic stroke recurrence was defined as a significant change in neurologic symptoms and signs accompanied by corresponding new discrete lesions on diffusion-weighted magnetic resonance images. Symptomatic hemorrhagic transformation was included as a safety outcome and was defined as a local or remote parenchymal hemorrhage type 2 on a post-treatment brain image combined with an increase of 4 points or more in the NIHSS score from baseline or with the occurrence of death [24].

### Statistical analysis

Study variables are expressed as mean ± SD, median (interquartile range, IQR), or number of patients (percentage) according to variable characteristics. Statin therapy was characterized according to the commencement of statin therapy, type, and dose (Additional file 1: Figure S1, S2 and Table S1). Baseline characteristics were compared according to the statin starting time using the Pearson *χ*^2^ test, ANOVA, and Kruskal-Wallis test when appropriate (Table 1).

**Table 1:** Comparison of baseline characteristics according to the statin starting time

Variables	Statin use			No use	*P* value*
	D1	D2	D ≥ 3		
	(*N* = 45)	(*N* = 70)	(*N* = 52)	(*N* = 170)	
Age, years, mean ± SD	67.6 ± 11.6	69.5 ± 12.0	68.0 ± 12.5	69.6 ± 12.7	0.69
Male sex	30 (66.7 %)	36 (51.4 %)	31 (59.6 %)	91 (53.5 %)	0.34
Time from onset to arrival, hours, mean ± SD	1.4 ± 1.3	1.6 ± 1.3 s	1.6 ± 1.4	1.5 ± 1.2	0.66
History of stroke	12 (26.7 %)	12 (17.1 %)	6 (11.5 %)	46 (27.1 %)	0.07
Hypertension	28 (62.2 %)	42 (60.0 %)	32 (61.5 %)	95 (55.9 %)	0.80
Diabetes mellitus	10 (22.2 %)	13 (18.6 %)	9 (17.3 %)	30 (17.6 %)	0.91
Atrial fibrillation	15 (33.3 %)	37 (52.9 %)	12 (23.1 %)	54 (31.8 %)	0.003
Premorbid statin use	8 (17.8 %)	10 (14.3 %)	6 (11.5 %)	15 (8.8 %)	0.33
Baseline NIHSS score, median (IQR)	15 (7–18)	11 (6-20)	12 (7.5–17)	15 (9–20)	0.14
Stroke subtype					0.002
LAA	20 (44.4 %)	27 (38.6 %)	21 (40.4 %)	42 (24.7 %)	
CE	13 (28.9 %)	39 (55.7 %)	22 (42.3 %)	96 (56.5 %)	
UD or OD	12 (26.7 %)	4 (5.7 %)	9 (17.3 %)	32 (18.8 %)	
Recanalization modality					0.007
IV-only	15 (33.3 %)	28 (40.0 %)	13 (25.0 %)	30 (17.6 %)	
IA-only	15 (33.3 %)	15 (21.4 %)	20 (38.5 %)	55 (32.4 %)	
Combined treatment	15 (33.3 %)	27 (38.6 %)	19 (36.5 %)	85 (50.0 %)	

Values represent number of patients (percentage) if not indicated

*P values were obtained by Pearson *χ* ^2^ test, ANOVA test, and Kruskal-Wallis test according to characteristics of variables

LAA is the abbreviation for large artery atherosclerosis; *CE* for cardioembolism, *UD* or *OD* for undetermined or other determined, *IV*-only for intravenous thrombolysis-only, *IA*-only for intra-arterial treatment-only, and *IQR* for interquartile range

**Fig. 1:**
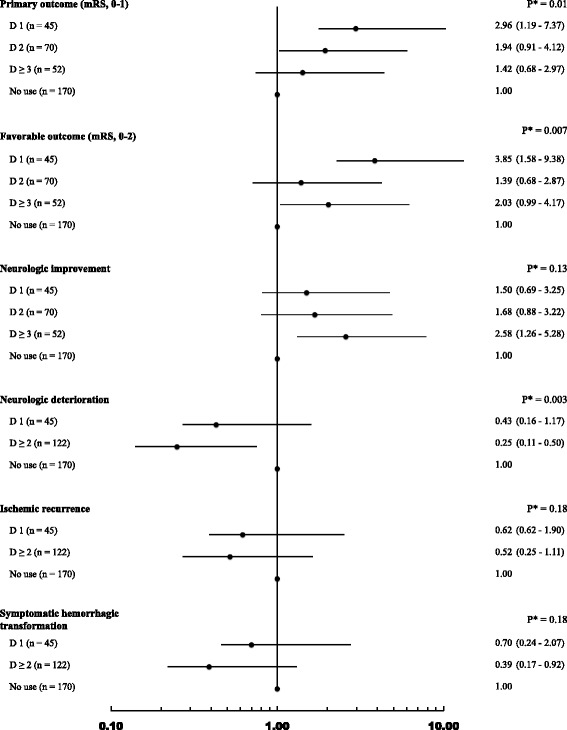
Adjusted odds ratios of the statin starting time with respect to various clinical outcomes. Statin starting time was defined as starting statin therapy at D1, D2, D ≥ 3, or no use, or as starting statin therapy at D1, D ≥ 2, and no use when event number of D2 or D ≥ 3 was less than 5. The adjusted odds ratios (circle) and 95 % confidence intervals (solid line) were estimated using multiple logistic regression models with adjustments for premorbid statin use, stroke history, atrial fibrillation, calendar year, stroke subtype, baseline NIHSS score, and recanalization modality. *Ps were calculated by log likelihood test for trend. mRS is the abbreviation for modified Rankin Score

Dose-response relationships between the statin starting time and the primary and secondary outcomes were evaluated using the Mantel-Haenszel test for trend (Table 2). Regarding multivariable analysis, adjusted odds ratios (ORs) of the statin starting time (D1, D2 and D ≥ 3) compared to NU were estimated for various outcomes, and dose-response relationships were characterized by likelihood ratio tests for trend (Fig. 1). In cases where the event number of D2 or D ≥ 3 was less than 5, the statin starting time was re-categorized into three groups: D1, D ≥ 2 and NU.

**Table 2:** Comparison of clinical outcomes according to the statin starting time

	Statin use			No use	*P* trend*
	D1	D2	D ≥ 3	(*N* = 170)	
	(*N* = 45)	(*N* = 70)	(*N* = 52)		
3-Month functional outcome					
Better primary outcome (mRS, 0-1)	19 (42.2 %)	26 (37.1 %)	18 (34.6 %)	38 (22.4 %)	0.002
Favorable outcome (mRS, 0-2)	26 (57.8 %)	30 (42.9 %)	29 (55.8 %)	57 (33.5 %)	0.004
Neurologic outcome during hospitalization					
Neurologic improvement^a^	24 (53.3 %)	39 (55.7 %)	38 (73.1 %)	84 (49.4 %)	0.87
Neurologic deterioration^b^	7 (15.6 %)	10 (14.3 %)	1 (1.9 %)	45 (26.5 %)	0.02
Ischemic recurrence	8 (17.8 %)	11 (15.7 %)	4 (7.7 %)	27 (15.9 %)	0.84
Symptomatic hemorrhagic transformation^c^	1 (2.2 %)	2 (2.9 %)	0 (0.0 %)	17 (10.0 %)	0.01

See footnotes of Table 1 for definitions and abbreviations

Values represent number of patients (percentage)

*P values were obtained by Mantel-Haenszel test for trend

^a^Neurologic improvement was defined as a decrease of ≥ 4 NIHSS score or a NIHSS score of 0 or 1 at discharge

^b^Neurologic deterioration was defined as an increase of ≥ 4 NIHSS score

^c^The definition of symptomatic hemorrhagic transformation was adopted from the SITS-MOST study [24]

Variables for adjustment were selected based on their p values (<0.2) and biological plausibility. To mitigate unmeasured confounding by recent advances in stroke care, calendar year was included in multivariable models (Additional file 1: Table S2). Because the number of patients with symptomatic hemorrhagic transformation was not enough to adjust all potential confounders at once, multivariable analyses were carried out using various sets of confounders as post-hoc analyses (Additional file 1: Table S3).

To investigate the heterogeneity of the early statin effect according to stroke subtype and recanalization modality, we performed subgroup analyses (cardioembolic vs. non-cardioembolic stroke and IV-only vs. IA-only vs. combined treatment) (Table 3 and 4). The statistical significance of the interaction between the statin starting time and stroke subtype or recanalization modality was examined in multivariable models. Finally, the associations of the statin dose with 3-month mRS 0–1 and symptomatic hemorrhagic transformation were analyzed (Additional file 1: Table S4 and S5). All statistical analyses were performed with SPSS version 18.0 (SPSS Inc., Chicago, IL). A two-sided p value of 0.05 was generally considered a minimum level of statistical significance.

**Table 3:** Subgroup analysis according to stroke subtype (cardioembolic stroke vs. non-cardioembolic stroke)

Outcomes	Statin use			No Use	*P* trend*
	D1	D2	D ≥ 3		
Cardioembolic stroke					
No. of patients	13	39	22	96	
Better primary outcome (mRS, 0-1)	3 (23.1 %)	13 (33.3 %)	7 (31.8 %)	19 (19.8 %)	0.19
Favorable outcome (mRS, 0-2)	4 (30.8 %)	16 (41.0 %)	9 (40.9 %)	32 (33.3 %)	0.63
Neurologic improvement	7 (53.8 %)	22 (56.4 %)	14 (63.6 %)	51 (53.1 %)	0.75
Neurologic deterioration	3 (23.1 %)	4 (10.3 %)	0 (0.0 %)	26 (27.1 %)	0.06
Ischemic recurrence	4 (30.8 %)	4 (10.3 %)	2 (9.1 %)	11 (11.5 %)	0.28
Symptomatic hemorrhagic transformation	0 (0.0 %)	1 (2.6 %)	0 (0.0 %)	10 (10.4 %)	0.04
Non-cardioembolic stroke^a^					
No. of patients	32	31	30	74	
Better primary outcome (mRS, 0-1)	16 (50.0 %)	13 (41.9 %)	11 (36.7 %)	19 (25.7 %)	0.01
Favorable outcome (mRS, 0-2)	22 (68.8 %)	14 (45.2 %)	20 (66.7 %)	25 (33.8 %)	0.003
Neurologic improvement	17 (53.1 %)	15 (54.8 %)	24 (80.0 %)	40 (54.1 %)	0.86
Neurologic deterioration	4 (12.5 %)	6 (19.4 %)	1 (3.3 %)	19 (25.7 %)	0.13
Ischemic recurrence	4 (12.5 %)	7 (22.6 %)	2 (6.7 %)	16 (21.6 %)	0.42
Symptomatic hemorrhagic transformation	1 (3.1 %)	1 (3.2 %)	0 (0.0 %)	7 (9.5 %)	0.14

See footnotes of Table 1 and 2 for definitions and abbreviations

Values represent number of patients (percentage)

*P values were calculated by Mantel-Haenszel test for trend

^a^Non-cardioembolism stroke consists of large artery atherosclerosis, stroke of other determined as well as undetermined etiology, according to the TOAST classification [18]

**Table 4:** Comparison of stroke outcomes according to the statin starting time and recanalization modalities

Outcome	Statin use			No Use	*P* trend*
	D1	D2	D ≥ 3		
IV-only					
No. of patients	15	28	13	30	
Better primary outcome (mRS, 0-1)	9 (60.0 %)	9 (32.1 %)	5 (38.5 %)	13 (43.3 %)	0.66
Favorable outcome (mRS, 0-2)	11 (73.3 %)	11 (39.3 %)	7 (53.8 %)	16 (46.7 %)	0.65
Neurologic improvement	8 (53.3 %)	15 (53.6 %)	8 (61.5 %)	13 (43.3 %)	0.95
Neurological deterioration	1 (6.7 %)	6 (21.4 %)	1 (7.7 %)	8 (26. 7 %)	0.20
Ischemic recurrence	2 (13.3 %)	6 (21.4 %)	2 (15.4 %)	4 (13.3 %)	0.71
Symptomatic hemorrhagic transformation	1 (6.7 %)	0 (0.0 %)	0 (0.0 %)	2 (6.7 %)	0.61
IA-only					
No. of patients	15	15	20	55	
Better primary outcome (mRS, 0-1)	3 (20.0 %)	5 (33.3 %)	5 (25.0 %)	9 (16.4 %)	0.38
Favorable outcome (mRS, 0-2)	4 (26.7 %)	5 (33.3 %)	9 (45.0 %)	16 (29.1 %)	0.96
Neurologic improvement	7 (46.7 %)	7 (46.7 %)	13 (65.0 %)	31 (56.4 %)	0.43
Neurological deterioration	5(33.3 %)	1 (6.7 %)	0 (0.0 %)	15 (27.3 %)	0.66
Ischemic recurrence	3 (20.0 %)	0 (0.0 %)	1 (5.0 %)	10 (18.2 %)	0.49
Symptomatic hemorrhagic transformation	0 (0.0 %)	1 (6.7 %)	0 (0.0 %)	7 (12.7 %)	0.08
Combined treatment					
No. of patients	15	27	19	85	
Better Primary outcome (mRS, 0-1)	7 (46.7 %)	12 (44.4 %)	8 (42.1 %)	16 (18.8 %)	0.002
Favorable outcome (mRS, 0-2)	11 (73.3 %)	14 (51.9 %)	13 (68.4 %)	25 (29.4 %)	<0.001
Neurologic improvement	9 (60.0 %)	17 (63.0 %)	17 (89.5 %)	43 (50.6 %)	0.23
Neurological deterioration	1 (6.7 %)	4 (11.1 %)	0 (0.0 %)	22 (25.9 %)	0.02
Ischemic recurrence	3 (20.0 %)	5 (18.5 %)	1 (5.3 %)	13 (15.3 %)	0.63
Symptomatic hemorrhagic transformation	0 (0.0 %)	1 (3.7 %)	0 (0.0 %)	8 (9.4 %)	0.09

See footnotes of Table 1 and 2 for definitions and abbreviations

Values represent number of patients (percentage)

*P values were calculated by the Mantel-Haenszel test for trend

## Results

Among 501 patients with acute ischemic stroke who were hospitalized within 6 h of onset and who underwent recanalization therapy, 345 had symptomatic stenosis or occlusion of major cerebral arteries at initial angiographic evaluation. Of those, eight patients were excluded due to no 3-month mRS, leaving a total of 337 patients included in the study. Mean age was 69.1 ± 12.4 years, with men comprising 55.8 %. Median baseline NIHSS score was 13 (IQR, 7–19). Recanalization therapy was IV-only in 25.5 %, IA-only in 31.2 %, and combined treatment in 43.3 %.

One-hundred sixty seven patients (49.6 %) received statin therapy during hospitalization. Analysis of the secular trends of the statin starting time demonstrated a gradual increase in the proportion of early users over time (Additional file 1: Figure S1). The proportion of patients receiving a statin at D1 was 0 % in 2004, but increased to 35 % in 2010. In total, 45 patients (13.4 %) started statin therapy at D1, 70 (20.8 %) started at D2, and 52 (15.4 %) started at D ≥ 3, while 170 (50.4 %) patients did not receive statin during hospitalization. Comparison of baseline characteristics revealed that atrial fibrillation, stroke subtype and recanalization modality were associated with the statin starting time (Table 1). With respect to the statin dose, 58.1 % of the statin users received a high dose of statin (40 mg or more of atorvastatin equivalent dose) (Additional file 1: Table S1); the proportion of high dose users began to increase in 2007 and leaped in 2009 (Additional file 1: Figure S2).

Comparisons between statin users vs. none users demonstrated better primary outcome (3-month mRS 0–1) in statin users (37.7 %) than in none users (22.4 %). Earlier use was positively associated with a better primary outcome (*P* for trend = 0.002) (Table 2). A similar trend was observed with respect to favorable outcome (mRS 0–2) (*P* for trend = 0.004). Earlier use was also associated with neurologic deterioration and symptomatic hemorrhagic transformation but not with neurologic improvement and ischemic recurrence (Table 2).

The dose-response relationship between the statin starting time and better primary outcome remained significant after adjustment for baseline NIHSS score, premorbid statin use, recanalization modality, atrial fibrillation, stroke subtype, calendar year, and stroke history (*P* for trend = 0.01). The odds of better primary outcome independently increased about 3-fold by commencement of statin therapy at D1 compared with no use (Additional file 1: Figure S1 and Table S2). Among the secondary outcomes, a dose-response relationship with the statin starting time was observed for favorable outcome and neurologic deterioration but not for neurologic improvement in multivariable analysis. With respect to symptomatic hemorrhagic transformation, all the multivariable analyses showed significant reduction of its odds in starting statin therapy at D ≥ 2 but not at D1 compared with no use (Additional file 1: Table S3).

Subgroup analysis according to stroke subtype revealed dose-response relationships between the statin starting time and better primary and more favorable outcomes in patients with non-cardioembolic stroke, and between the statin starting time and symptomatic hemorrhagic transformation in patients with cardioembolic stroke (Table 3). Interaction analysis with adjustments for potential confounders did not show any statistically significant heterogeneity of the early statin effect between cardioembolic and non-cardioembolic stroke (*P* for interaction = 0.97).

Subgroup analysis according to recanalization modality demonstrated a clear dose-response relationship between the statin starting time and better primary/more favorable outcome and neurologic deterioration in patients who received combined treatment but not in those who received IV-only or IA-only (Table 4). However, there was no statistically significant interaction between the statin starting time and recanalization modality in the multivariable model (*P* for interaction = 0.26).

With regard to statin dose, a higher dose tended to increase the odds of better primary outcome compared to no use (*p* for trend = 0.07) while significantly reducing the odds of symptomatic hemorrhagic transformation (*p* for trend = 0.03) (Additional file 1: Table S4 and S5).

## Discussion

Our study demonstrates that early use of statin may improve functional outcome in patients with acute symptomatic steno-occlusion of major cerebral arteries treated with recanalization therapy. Our findings also highlight that the use of statin is most effective when started on the first day after recanalization therapy. Furthermore, the effect of statin therapy did not differ with regard to stroke subtype and recanalization modality. As for safety, earlier starting or higher dose of statin therapy did not increase the risk of symptomatic hemorrhagic transformation but rather decreased this safety metric.

While the efficacy of early statin use after acute coronary syndrome has been reported [25, 26], the use of statin in patients with acute ischemic stroke and treated with recanalization therapy has been investigated on a limited scale. Recently, the THRombolysis and Statins (THRaST) study showed that statin use in the acute phase (within 72 h) after intravenous thrombolysis might positively influence short- and long-term outcomes by increasing neurologic improvement (OR, 1.68) and favorable functional outcome (OR, 1.63), and by reducing neurologic deterioration (OR, 0.31) and death (OR 0.48) [15]. However, several potential limitations of this data should be considered. First, the THRaST study did not determine dose-response relationships between the statin starting time and clinical outcomes. Second, 22 % of the THRaST participants had atrial fibrillation, but the study did not address the potential efficacy and safety concerns related to early use of statin in cardioembolic stroke, a distinct stroke subtype in terms of stroke mechanism and risk of hemorrhagic transformation. Lastly, the THRaST study targeted patients treated with intravenous thrombolysis only, but did not target those treated with IA-only or combined treatment. The proportion of patients treated by endovascular approaches is not negligible nowadays. In our study, approximately 75 % of patients who received recanalization therapy were treated with IA-only or combined treatment.

Our study addressed three points: First, starting statin at the first day after recanalization therapy increased the odds of a better functional outcome by approximately three-fold and this beneficial effect decreased gradually over time. The proportion of patients who started statin therapy at the first hospital day was 35 % in 2010 and 21 % in 2011 (Additional file 1: Figure S1) despite the overt increase of statin users since 2007. Therefore, we may have a window of opportunity to improve outcome by commencing statin therapy at this early time-point. Second, our study showed that early statin therapy did not increase, and that it rather decreased the risk of symptomatic hemorrhagic transformation in patients with cardioembolic stroke; also, the beneficial effect of early statin therapy was not altered by stroke subtype (cardioembolic vs. non-cardioembolic). Since early differentiation of cardioembolic stroke from other stroke subtypes may not be feasible in most practices [27], this study highlights the safety and potential benefit of early statin use in patients with acute symptomatic steno-occlusion who are treated with recanalization therapy and whose stroke mechanisms were obscure at presentation. Third, with respect to recanalization modality, there was no significant heterogeneity of the early statin effect, although a clear benefit was observed in the combined treatment group. The relatively small sample size of this study for subgroup analysis suggests the need of a study with a larger sample size.

There has been some skepticism regarding the increased risk of symptomatic hemorrhagic transformation associated with statin therapy after IV and IA thrombolysis [28, 29]. The results of THRaST and our study taken together suggest that early use of statin after thrombolysis does not increase, and may reduce the risk of hemorrhagic transformation. Furthermore, our study demonstrates the dose-response relationship between statin dose and the prevention of symptomatic hemorrhagic transformation.

Analysis of short-term clinical outcomes during hospitalization suggests that early statin therapy might potentially prevent neurologic deterioration and symptomatic hemorrhagic transformation. These findings support the hypothesis that statin therapy may protect neuronal cells from reperfusion injury and major symptomatic brain arteries from re-occlusion [30]. Practically speaking, the risk of neurologic deterioration is time-dependent, [31], and starting statin therapy immediately after or even before recanalization therapy may maximize the statin effect.

We note several potential study limitations. First, the study was conducted in a single community-based hospital, and the study participants were identified in a retrospective manner. Although the patients were enrolled from the prospective stroke registry database and the study outcomes were captured prospectively, we may be dealing with patients who do not represent the community-at large, and thus, our findings may not be representative of other populations. Furthermore, considering the observational nature of our study design, residual or unmeasured confounding effects could be introduced and, hence, our results are not free of risk of inevitable biases despite adjusting for potential confounders through modeling. For example, the rate of statin use increased over time, therefore improvement of outcome by statin use may be attributed to improvement of stroke management over time, although calendar year was included in the multivariable models to adjust for that kind of confounding effect. Second, subgroups in our analysis were occasionally small and therefore our findings based on multiple comparisons could be because of chance. However, the subgroup analysis according to recanalization modality suggested the possibility of effect modification, although this was statistically not significant. Residual confounding by recanalization modality also cannot be excluded. Third, all forms and doses of statins were converted to atorvastatin equivalents as though there was one type of statin drug and the statin starting time was not considered in the analysis of statin dose. The heterogeneity of the early statin effect according to statin form and dose should be considered in a study with a larger sample size. Forth, there were concerns that physician might use statin at earlier time in patients with successful recanalization and it would affect the result. Because we did not analyze the association between successful recanalization and statin starting time in this study, that problem could not be solved exactly. However, IA-only and combined treatment groups showed similar or low rates of statin use at the first day of admission (14.2 % and 10.3 %) compared with the IV-only group (17.4 %) although higher recanalization rates were expected in the former groups, and these features might mitigate our concerns. Finally, it should be clearly noted that less frequent symptomatic hemorrhagic transformation and neurologic deterioration in statin users might be attributed to preferential underuse of statin in patients with high risk of hemorrhagic transformation or worse prognosis. A randomized clinical trial would be a more robust setting for answering the questions we posed.

## Conclusion

Early use of statin after recanalization therapy may improve functional outcome without increasing intracranial hemorrhage in patients with symptomatic steno-occlusion of major cerebral arteries. The effect was noted in all stroke subtypes and regardless of recanalization modality.

## Additional file

Additional file 1: Figure S1. Secular trends of the statin starting time in acute ischemic stroke. Statin starting time was defined by the following groups: first day (D1, dark grey box), second day (D2, grey box), third day or later (D ≥ 3, white grey box) of hospitalization, and no use (white box). **Figure S2.** Secular trend of statin dose in acute ischemic stroke. Statin dose was converted to an atorvastatin equivalent dose ≥ 40 mg (dark grey box), atorvastatin equivalent dose < 40 mg (grey white box), and no use (white box). **Table S1.** Summary of forms and doses of statin used during hospitalization. **Table S2.** Multivariable logistic regression analysis: the statin starting time and 3-month mRS 0-1. **Table S3.** Multivariable analyses using various sets of confounders: the statin starting time and symptomatic hemorrhagic transformation. **Table S4.** Multivariable logistic regression analysis: the statin dose and 3-month mRS 0-1. **Table S5.** Multivariable logistic regression analysis: the statin dose and symptomatic hemorrhagic transformation.
