# Supplementary material for: Early statin use in ischemic stroke patients treated with recanalization therapy: retrospective observational study
Source: BMC Neurol. 2015 Jul 30;15:122. doi: 10.1186/s12883-015-0367-4 (PMC4520147; doi:10.1186/s12883-015-0367-4)
Supplement: Additional file 1: Figure S1. — Secular trends of the statin starting time in acute ischemic stroke. Statin starting time was defined by the following groups: first day (D1, dark grey box), second day (D2, grey box), third day or later (D ≥ 3, white grey box) of hospitalization, and no use (white box). Figure S2. Secular trend of statin dose in acute ischemic stroke. Statin dose was converted to an atorvastatin equivalent dose ≥ 40 mg (dark grey box), atorvastatin equivalent dose < 40 mg (grey white box), and no use (white box). Table S1. Summary of forms and doses of statin used during hospitalization. Table S2. Multivariable logistic regression analysis: the statin starting time and 3-month mRS 0-1. Table S3. Multivariable analyses using various sets of confounders: the statin starting time and symptomatic hemorrhagic transformation. Table S4. Multivariable logistic regression analysis: the statin dose and 3-month mRS 0-1. Table S5. Multivariable logistic regression analysis: the statin dose and symptomatic hemorrhagic transformation. [file 12883_2015_367_MOESM1_ESM.docx]

**Supplemental Figure Legends**

Supplemental Figure 1. Secular trends of the statin starting time in acute ischemic stroke

Statin starting time was defined by the following groups: first day (D1, dark grey box), second day (D2, grey box), third day or later (D≥3, white grey box) of hospitalization, and no use (white box).

Supplemental Figure 2. Secular trend of statin dose in acute ischemic stroke

Statin dose was converted to an atorvastatin equivalent dose ≥ 40mg (dark grey box), atorvastatin equivalent dose < 40mg (grey white box), and no use (white box).

**Supplemental Tables**

Supplemental Table 1. Summary of forms and doses of statin used during hospitalization

| Atorvastatin equivalent dose* | No. of patients (%) | Pitavastatin | Simvastatin | Pravastatin | Rosuvastatin | Atorvastatin |
| --- | --- | --- | --- | --- | --- | --- |
| 2·5mg | 7 (4.2%) |  |  | 10mg (7) |  |  |
| 10mg | 54 (32.3%) | 2mg (7) | 20mg (4) | 40mg (1) |  | 10mg (42) |
| 20mg | 9 (5.4%) |  |  |  | 5mg (1) | 20mg (8) |
| 40mg | 33 (19.8%) |  |  |  | 10mg (6) | 40mg (27) |
| 80mg | 64 (38.3%) |  |  |  |  | 80mg (64) |

Values represent statin dose in milligrams or the number of patients if not indicated. Based on comparative efficacy of various statins on lipid fractions, doses of various statin forms were substituted with atorvastatin equivalent dose.

Supplemental Table 2. Multivariable logistic regression analysis: the statin starting time and 3-month mRS 0-1

| Variables | Adjusted odds ratio | 95% confidence interval |
| --- | --- | --- |
| History of stroke | 0.39 | 0.19-0.82 |
| Atrial fibrillation | 0.53 | 0.23-1.23 |
| Premorbid statin use | 3.57 | 1.61-8.08 |
| Baseline NIHSS score | 0.89 | 0.86-0.93 |
| Calendar year | 0.92 | 0.77-1.10 |
| Recanalization modalities |  |  |
| IV-only | 1 |  |
| IA-only | 0.44 | 0.21-0.89 |
| Combined treatment | 0.85 | 0.45-1.58 |
| Stroke subtype |  |  |
| LAA | 1 |  |
| CE | 1.05 | 0.49-2.24 |
| OD or UD | 1.08 | 0.49-2.38 |
| Statin starting time |  |  |
| No use | 1 |  |
| D≥3 | 1.42 | 0.67-2.97 |
| D2 | 1.94 | 0.91-4.12 |
| D1 | 2.96 | 1.19-7.37 |

See footnote of Table 1 and 2 for definitions and abbreviations.

Supplemental Table 3. Multivariable analyses using various sets of confounders: the statin starting time and symptomatic hemorrhagic transformation

|  | No use | D≥2 | D1 |
| --- | --- | --- | --- |
| Crude | 1 | 0.40 (0.18-0.89) | 0.78 (0.30-2.02) |
| Model A | 1 | 0.38 (0.17-0.88) | 0.70 (0.25-1.95) |
| Model B | 1 | 0.38 (0.17-0.88) | 0.67 (0.23-1.89) |
| Model C | 1 | 0.41 (0.18-0.96) | 0.71 (0.25-2.04) |
| Model D | 1 | 0.42 (0.18-0.96) | 0.72 (0.25-2.11) |
| Model E | 1 | 0.41 (0.17-0.95) | 0.71 (0.24-2.11) |

See footnotes of Figure 1 for definitions and abbreviations.

Values are odds ratios (95% confidence intervals).

Model A was adjusted for the calendar year; Model B was adjusted for the calendar year and recanalization modalities; Model C was adjusted for the calendar year, recanalization modalities, and baseline NIHSS scores; Model D was adjusted for the calendar year, recanalization modalities, baseline NIHSS scores, and atrial fibrillation; and Model E was adjusted for the calendar year, recanalization modalities, baseline NIHSS scores, atrial fibrillation, and stroke subtypes.

Supplemental Table 4. Multivariable logistic regression analysis: the statin dose and 3-month mRS 0-1

| Variables | Adjusted odds ratio | 95% confidence interval |
| --- | --- | --- |
| History of stroke | 0.41 | 0.20-0.86 |
| Atrial fibrillation | 0.53 | 0.23-1.21 |
| Premorbid statin use | 3.53 | 1.57-7.95 |
| Baseline NIHSS score | 0.90 | 0.86-0.93 |
| Recanalization modalities |  |  |
| IV-only | 1 |  |
| IA-only | 0.44 | 0.21-0.89 |
| Combined treatment | 0.83 | 0.45-1.56 |
| Stroke subtype |  |  |
| LAA | 1 |  |
| CE | 0.97 | 0.46-2.06 |
| OD or UD | 1.06 | 0.49-2.32 |
| Statin dose^a^ |  |  |
| No use | 1 |  |
| Low dose (N=70) | 1.79 | 0.92-3.48 |
| High dose (N=97)* | 1.85 | 0.86-3.96 |
| P for trend^†^ = 0.068 |  |  |

See footnotes of Table 1 and 2 for definitions and abbreviations.

*High dose was defined as 40mg of atorvastatin equivalent dose or more during hospitalization, and low dose was defined as less than 40mg.

^†^P was calculated by log likelihood test for trend.

.

Supplemental Table 5. Multivariable logistic regression analysis: the statin dose and symptomatic hemorrhagic transformation

| Variables | Adjusted odds ratio | 95% confidence interval |
| --- | --- | --- |
| History of stroke | 0.86 | 0.38-1.90 |
| Atrial fibrillation | 1.01 | 0.37-2.80 |
| Premorbid statin use | 1.28 | 0.44-3.70 |
| Baseline NIHSS score | 1.05 | 1.00-1.10 |
| Calendar year | 1.12 | 0.89-1.40 |
| Recanalization modalities |  |  |
| IV-only | 1 |  |
| IA-only | 1.73 | 0.68-4.43 |
| Combined treatment | 1.02 | 0.44-3.70 |
| Stroke subtype |  |  |
| LAA | 1 |  |
| CE | 0.90 | 0.34-2.36 |
| OD or UD | 0.78 | 0.28-2.18 |
| Statin dose |  |  |
| No use | 1 |  |
| Low dose (N=70) | 0.60 | 0.24-1.50 |
| High dose (N=97) | 0.36 | 0.13-0.94 |
| P for trend = 0.028 | 0.86 | 0.38-1.90 |

See footnote Table 1 and 2 and Supplemental Table 4 for definitions and abbreviations.
